# Two-stage revision anterior cruciate ligament reconstruction reduces failure risk but leads to lesser clinical outcomes than single-stage revision after primary anterior cruciate ligament graft failure: a retrospective cohort study

**DOI:** 10.1186/s43019-024-00257-y

**Published:** 2025-01-15

**Authors:** Anna M. Ifarraguerri, George D. Graham, Alexander B. White, Alexander N. Berk, Kennedy K. Gachigi, Patrick N. Siparsky, David P. Trofa, Dana P. Piasecki, James E. Fleischli, Bryan M. Saltzman

**Affiliations:** 1 OrthoCarolina – Sports Medicine Center, 1915 Randolph Road, Charlotte, NC 28207 USA; 2 OrthoCarolina Research Institute, 2001 Vail Ave, #300, Charlotte, NC 28207 USA; 3 Atrium Health – Musculoskeletal Institute, 1320 Scott Ave, Charlotte, NC 28203 USA; 4 Department of Orthopaedics, New York Presbyterian, Columbia University Medical Center, 622 West 168th St, PH 111-1130, New York, NY 10032 USA; 5 IU Health Physicians Orthopedics & Sports Medicine, 1801 N Senate Ave, Indianapolis, IN 46202 USA; 6 Department of Orthopaedic Surgery, Indiana University School of Medicine, 550 N. University Blvd. 6201, Indianapolis, IN 46202 USA

**Keywords:** Revision ACLR, Two-stage, Outcomes, Single-stage

## Abstract

**Background:**

There are no studies that compare the outcomes and complications of single-versus two-stage revision anterior cruciate ligament reconstruction (ACLR) after primary ACLR failure. This purpose of this study is to examine clinical and functional outcomes and complications associated with single and two-stage revision ACLR after primary ACLR failure.

**Methods:**

All patients who underwent single or two-stage revision ACLR after primary ACLR failure between 2012 and 2021 with a minimum of a 2 year follow-up were included. Patients were excluded if they were not treated at our single academic institution, had inadequate follow-up, or had incomplete medical records. Revision intraoperative data, concomitant injuries, and complications were collected by chart review. Return to sport, numerical pain rating scale (NPRS) score, Knee injury and Osteoarthritis Outcome Score (KOOS), and Veteran Rands 12-item health survey (VR-12 scores) were collected.

**Results:**

The final analysis included 176 patients. A total of 147 (83.5%) had a single-stage revision ACLR (87 male, 60 female), and 29 (16.5%) had a two-stage revision ACLR (13 male, 16 female). Two-stage revision ACLR was significantly associated with anterior knee pain [odds ratio (OR) 4.36; 95% confidence interval (CI) 1.5 to 12.65; *P* = 0.007] but with lower failure rates (OR 0.12, 95% CI 0.02 to 0.9; *P* = 0.04). On multivariate analysis, a two-stage revision ACLR reduced the risk of graft failure by 85% (OR 0.15; 95% CI 0.02 to 1.17; *P* = 0.07). Two-stage revision ACLR was significantly associated with a lower KOOS pain score (OR −11.7; 95% CI −22.35 to −1.04; *P* = 0.031), KOOS symptoms score (OR −17.11; 95% CI −28.85 to −5.36; *P* = 0.004), KOOS Activities of Daily Living (ADL) score (OR −11.15; 95% CI −21.71 to −0.59; *P* = 0.039) and Veterans RAND 12-Item Health Survey (VR-12) physical component score (OR −9.99; 95% CI −15.77 to −4.22; *P* = 0.001).

**Conclusions:**

The clinical outcomes and subjective patient scores significantly differed between the single-stage and two-stage revision ACLR after primary ACLR failure. Patients with a two-stage revision ACLR had a significantly reduced risk of revision graft failure but higher rates of postoperative anterior knee pain, lower pain scores, and lesser knee functional scores than single-stage revision patients.

**Study design:**

Retrospective cohort study; level of evidence, 3

## Introduction

Anterior cruciate ligament (ACL) tears are one of the most common sports-related injuries [1] and have an increasing incidence of 68.6 per 100,000 person-years [2]. ACL reconstruction (ACLR) is the gold standard for patients to return to their preinjury sport level [3–5]; however, there is a 5.2% rate of graft failure [6], which increases to 34.2% for high-risk cohorts such as younger athletes [7]. Paralleling the rise in ACL rupture is an increase in the number of graft ruptures and failures, with rates estimated to be 6.2% and 11.9%, respectively [8, 9].

The etiology of graft failure is most commonly traumatic but can also occur due to technical error, lower extremity malalignment, and biological failure of graft incorporation [10]. Additional risk factors for graft failure include the type of graft, lateral meniscal deficiency, younger patient age, early return to sport, and contact mechanism of the initial injury [11–16].

Revision ACLR outcomes are less successful than primary ACLR in terms of functional scores, patient satisfaction, and risk of developing knee osteoarthritis [7, 17]. However, outcomes of revision ACLR tend to be preferable to conservative treatment after graft failure [12, 18]. When revision ACLR is undertaken, it can be performed as either a single-stage or two-stage procedure. Single-stage ACLR is performed when there is anatomic tunnel placement without evidence of excessive widening or osteolysis and when the new tunnels can be placed without intersecting the previous ones [19]. When these parameters are unmet, a two-stage revision is carried out in which the initial tunnels are filled with a bone graft, followed by reconstruction after sufficient graft incorporation.

A limited number of studies compare the outcomes of a single- versus two-stage revision ACLR. A 2017 study by Mitchell et al. directly comparing single- and two-stage revision ACLR concluded no significant difference in failure rates, objective outcomes, and subjective patient scores at a mean of 3 years [20]. However, the study included both initial and subsequent ACLR revisions as they did not exclude patients with a previous history of ACLR revision surgery. A study that utilized the Danish Knee Ligament Reconstruction Registry found no significant differences in failure rates between single-stage and two-stage revision ACLR with 2 year follow-up, but no patient-reported outcomes were reported [21]. Additionally, no studies compare the complications between single- or two-stage revision ACLR after primary ACLR failure. Thus, the outcomes and complications after primary single-stage or two-stage revision ACLR still need to be clarified. It is hypothesized that patients undergoing single-stage revision ACLR will have improved outcomes, fewer complications, and improved graft survival relative to those undergoing a two-stage procedure. This study examines clinical and functional outcomes and complications associated with single- and two-stage revision ACLR after primary ACLR failure.

## METHODS

### Study design

A retrospective cohort study design was used to compare the outcomes of patients who underwent a single-stage or a two-stage revision ACLR after primary ACLR failure.

### Patient identification

After obtaining approval from the institutional review board (IRB00089183), we performed a query of the administrative database at a single academic institution from 2012 to 2021 using the Current Procedural Terminology code 29888 (ACL reconstruction). Operative reports were then reviewed to distinguish between primary or revision ACLR patients. Patients 14 years of age and older who underwent their first revision ACLR were identified, and all patients with a minimum of a 2 year follow-up were considered for inclusion. Exclusion criteria included patients treated outside our academic institution, inadequate follow-up, or incomplete medical records.

### Surgical technique

Indications for each revision procedure were collected. Patients underwent two-stage revision ACLR under the following circumstances: tibial/femoral tunnel widening > 12 mm, mispositioned femoral tunnel, and inability to drill new tunnels due to interference with previously drilled tunnels. All patients were treated by fellowship-trained sports medicine orthopedic surgeons at our single academic institution. Overall patient health, including vascular status and ability to adhere to postoperative rehabilitation protocols, was assessed individually and used to determine patient appropriateness for surgery. Surgical approaches and graft types were not standardized for this study, but operative data were collected to control this in the analysis.

### Postoperative rehabilitation and follow-up

The postoperative rehabilitation varied by surgeon and concomitant injuries but followed best practice guidelines. After the revision ACLR procedure, patients were allowed to weight bear with crutches as tolerated at the physician’s discretion for 4 weeks, and to start physical therapy immediately after surgery. Patients could return to pivoting and cutting at a minimum of 6 months postoperatively at the discretion of the surgeon and physical therapist. Patients who underwent a staged bone-grafting procedure could weight bear as tolerated at the surgeons discretion with crutches for 2 weeks and remain in a playmaker brace until revision surgery. Patients who underwent staged meniscal or osteotomy operations followed the appropriate standard rehabilitation protocols for that procedure. Routine follow-up was scheduled at 1–2 weeks, 6 weeks, 3 months, 6 months, 9 months, or more as needed. Equipment availability did not differ across rehabilitation facilities.

### Data collection

Clinical and operative notes of all eligible patients were reviewed, and demographic, operative, and postoperative data were obtained. Surgical variables included graft type, tunnel drilling method, and graft fixation type. All data were extracted from postoperative clinic notes as documented by the treating surgeon. Concomitant injuries and procedures for each reconstruction were collected. We assessed postoperative complications, including anterior knee pain, superficial skin infection (SSI)/wound dehiscence, deep infection, stiffness/arthrofibrosis, symptomatic hardware, venous thromboembolism (VTE), and graft failure.

For this study, stiffness was defined as any medical or surgical treatment for a restricted range of motion, including corticosteroids (oral or intra-articular). Superficial wound infection or wound dehiscence was identified by the prescription of antibiotics in response to documented wound concerns or a clear description of gross wound abnormalities. All episodes of deep infection required a return to the operating room for irrigation and debridement. Symptomatic hardware was considered as patient-reported pain at the hardware site or procedure of hardware removal for pain at the hardware site. Graft failure was defined as graft rerupture, additional ACLR revision procedure, pivot shift 3+ , positive Lachman exam, or evidence of graft failure on magnetic resonance imaging (MRI). Study data were collected and managed using REDCap (Vanderbilt University), a secure web-based software platform hosted at OrthoCarolina Research Institute [22].

### Patient reported outcomes

Patient-reported outcome (PRO) questionnaires were emailed to patients or collected over the phone to obtain postoperative outcome scores. Patients completed subjective questionnaires at least 2 years postoperatively, including numerical pain rating scale (NPRS), return to play, Knee Injury and Osteoarthritis Outcome Score (KOOS), and the Veterans RAND 12-Item Health Survey (VR-12). Patients were contacted up to three times to request participation in the surveys but were not excluded if they did not complete the PROs.

### Statistical analysis

Descriptive statistics were calculated for all continuous and categorical variables. Continuous variables are reported as mean ± standard deviation (SD), and categorical variables as frequencies with percentages. A bivariate analysis of single-stage versus two-stage was performed using the Wald chi-square (*χ*^2^) test to analyze categorical variables, and a *t*-test was used to test the difference in means for continuous variables. *P* ≤ 0.05 was considered statistically significant. An additional matched analysis of the two cohorts matched by sex, age, and body mass index (BMI) was performed. The matched patients’ demographics, intraoperative data, and complications were analyzed. PROs were not included in the matched analysis, as the matching removed many patients who completed the PROs from the analysis.

Multivariate analysis to predict revision graft failure was performed using a binary logistic regression model. Significant values from the non-matched and matched bivariate analyses were included in the multivariate model. Risk factors with a significant likelihood ratio (*P* ≤ 0.1) in the predictive model were included in the final model.

All statistical analysis was performed using SAS Version 9.4 (SAS Institute). An a priori power analysis was not performed because all eligible patients were included in the study.

## Results

### Demographics

**Fig. 1:**
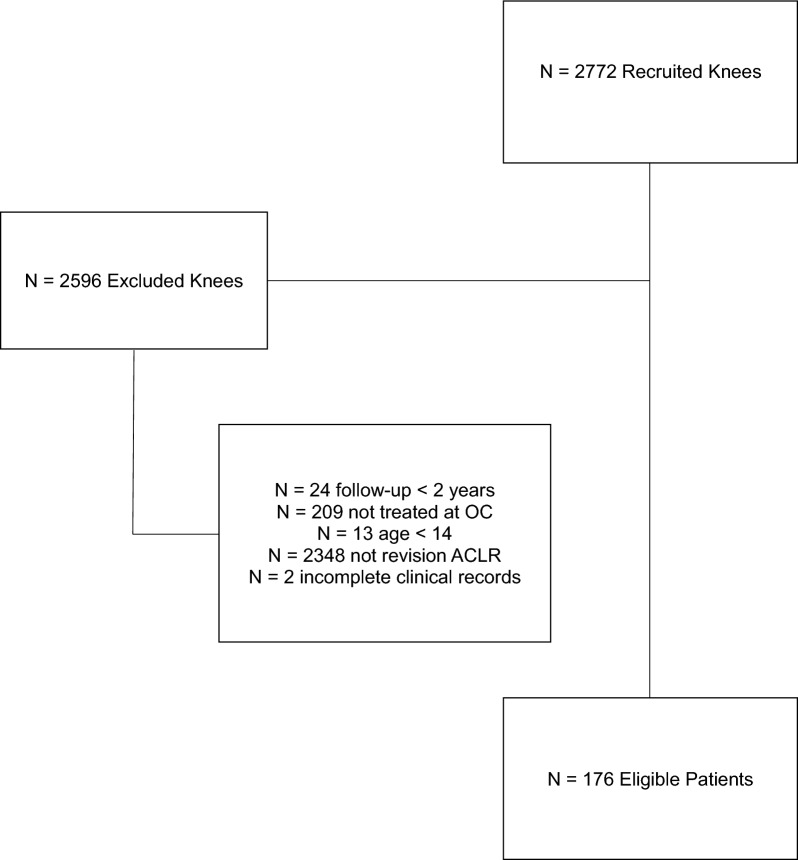
Consolidated Standards of Reporting Trials (CONSORT) diagram for outcomes of single-stage versus two-stage revision ACLR

After inclusion and exclusion criteria were applied, 176 patients were included in the final analysis (Fig. 1). A total of 147 (83.5%) patients had a single-stage revision ACLR procedure, and 29 (16.5%) had a two-stage revision ACLR after primary ACLR failure. Among the single-stage cohort, 87 (59.2%) were male, and 60 (40.8%) were female (Table 1). In the two-stage cohort, 13 (44.8%) were male and 16 (55.2%) were female. The mean age for the single-stage cohort was 25.9 ± 10.9 years and 26.2 ± 10.2 years for the two-stage cohort (OR 0.27; 95% CI −4.01 to 4.56; *P* = 0.90). The single-stage cohort had a mean body mass index (BMI) of 26 ± 5.5 kg/m^2^, and the two-stage cohort had a mean BMI of 28.3 ± 6.3 kg/m^2^ (OR 2.2; 95% CI −0.05 to 4.46; *P* = 0.056). The time from primary ACLR to revision ACLR was 43.1 ± 47.3 months for patients who underwent a single-stage revision ACLR and 41.2 ± 30.2 months for the two-stage revision ACLR patients (OR 4; 95% CI −20.09 to 16.41; *P* = 0.843).

**Table 1:** Baseline demographics^a^

	One-stage (*n* = 147)	Two-stage (*n* = 29)	OR (95% CI)	*P* value
Male	87 (59.2%)	13 (44.8%)	1.78 (0.8 to 3.98)	0.157
Female	60 (40.8%)	16 (55.2%)		
Age at surgery, years	25.9 (10.9)	26.2 (10.2)	0.27 (−4.01 to 4.56)	0.9
BMI, kg/m^2^	26 (5.5)	28.3 (6.3)	2.2 (−0.05 to 4.46)	0.056
Time primary to revision ACLR, months	43.1 (47.3)	41.2 (30.2)	4 (−20.09 to 16.41)	0.843

^a^Data are presented as mean (standard deviation) or *n* (%). Boldface *P* values indicate statistically significant difference between groups (*P* ≤ 0.05)

*ACLR* anterior cruciate ligament reconstruction, *BMI* body mass index, *CI* confidence interval, *OR* odds ratio

### Intraoperative

On bivariate analysis, there was no significant difference between the use of autograft tendon versus allograft in single-stage (57.8%) and two-stage (79.3%) revision ACLR (OR 2.84; 95% CI 0.92 to 8.75; *P* = 0.069) (Table 2). There were no significant differences in graft tendon type between the revision cohorts. The use of a metal interference screw for tibial graft fixation was associated with two-stage revision ACLR (65.5%) over single-stage (42.2%) revision ACLR (OR 2.6; 95% CI 1.13 to 5.99; *p* = 0.024). Similarly, there was a higher association in the rate of metal interference screw for femoral graft fixation between single-stage (42.9%) and two-stage (65.5%) revision ACLR (OR 2.53; 95% CI 1.1 to 5.82; *P* = 0.029). Regarding the femoral tunnel drilling approach, there was a higher frequency in the use of the hybrid transtibial (HTT) approach for two-stage revision ACLR (55.2%) compared with single-stage revision ACLR (15.6%) (OR 27.83; 95% CI 3.46 to 223.71; *P* = 0.002) (Table 2).

**Table 2:** Revision intraoperative data^a^

	One-stage (*n* = 147)	Two-stage (*n* = 29)	OR (95% CI)	*P* value
Allograft	42 (28.6%)	4 (13.8%)	Referent	Referent
Allograft augmentation,	18 (12.2%)	1 (3.4%)	0.58 (0.06 to 5.59)	0.64
Autograft	85 (57.8%)	23 (79.3%)	2.84 (0.92 to 8.75)	0.069
Hamstring tendon	18 (12.2%)	3 (10.3%)	Referent	Referent
Patellar tendon	84 (57.1%)	22 (75.9%)	1.57 (0.42 to 5.82)	0.499
Quad tendon	5 (3.4%)	1 (3.4%)	0.76 (0.08 to 6.88)	0.81
Other	18 (12.2%)	2 (6.9%)	0.42 (0.09 to 1.97)	0.273
Metal interference screw, tibial	62 (42.2%)	19 (65.5%)	2.6 (1.13 to 5.99)	**0.024**
Bioabsorbable interference screw, tibial	44 (29.9%)	6 (20.7%)	0.61 (0.23 to 1.6)	0.317
Washer, tibial	41 (27.9%)	2 (6.9%)	0.19 (0.04 to 0.84)	**0.029**
Staples, tibial	62 (42.2%)	19 (65.5%)	2.45 (0.7 to 8.58)	0.16
Loop cortical suspensory device, tibial	9 (6.1%)	4 (13.8%)	0.54 (0.21 to 1.41)	0.207
Suture post, tibial	19 (12.9%)	3 (10.3%)	0.78 (0.21 to 2.82)	0.702
Hybrid, tibial	42 (28.6%)	6 (20.7%)	0.65 (0.25 to 1.72)	0.386
Metal interference screw, femoral	63 (42.9%)	19 (65.5%)	2.53 (1.1 to 5.82)	**0.029**
Bioabsorbable interference screw, femoral	39 (26.5%)	4 (13.8%)	0.44 (0.14 to 1.35)	0.153
Button, femoral	12 (8.2%)	3 (10.3%)	1.3 (0.34 to 4.92)	0.701
Washer, femoral	8 (5.4%)	2 (6.9%)	1.29 (0.26 to 6.4)	0.758
Suture post, femoral	3 (2%)	1 (3.4%)	1.71 (0.17 to 17.08)	0.646
Hybrid, femoral	18 (12.2%)	1 (3.4%)	0.26 (0.03 to 2)	0.194
Transtibial	40 (27.2%)	1 (3.4%)	Referent	Referent
Transportal	43 (29.3%)	5 (17.2%)	4.65 (0.52 to 41.55)	0.169
Hybrid transtibial	23 (15.6%)	16 (55.2%)	27.83 (3.46 to 223.71)	**0.002**
Outside-in	9 (6.1%)	2 (6.9%)	8.89 (0.72 to 109.05)	0.088
Other femoral tunnel drilling	19 (12.9%)	2 (6.9%)	4.21 (0.36 to 49.37)	0.252
Tibial tunnel positioning, mispositioned	28 (19%)	14 (48.3%)	4.91 (2.01 to 11.98)	** < 0.001**
Femoral tunnel positioning, mispositioned	46 (31.3%)	15 (51.7%)	3.26 (1.33 to 8.02)	**0.01**

^a^Data are presented as mean (standard deviation) or *n* (%). Boldface *P* values indicate statistically significant difference between groups (*P* ≤ 0.05)

*CI* confidence interval, *OR* odds ratio

Patients who had a two-stage procedure had a significant association with concomitant meniscus repair (OR 2.6; 95% CI 1.1 to 6.11; *P* = 0.029), concomitant other ligament reconstruction (OR 7.56; 95% CI 2.87 to 19.92;* P* < 0.001), and simultaneous high tibial osteotomy (HTO) (OR 23.36; 95% CI 2.51 to 217.68; *P* = 0.006) (Table 3) compared with patients who had a single-stage revision ACLR. No significant differences existed in the rates of chondral injuries or procedures, meniscectomies, or anterolateral ligament augmentation between the single-stage and two-stage revision cohorts (Table 3).

**Table 3:** Revision concomitant injuries and procedures^a^

	One-stage (*n* = 147)	Two-stage (*n* = 29)	OR (95% CI)	*P* value
Chondral defect	42 (28.6%)	10 (34.5%)	1.32 (0.57 to 3.06)	0.525
Meniscal lesion	88 (59.9%)	20 (69%)	1.49 (0.63 to 3.5)	0.36
Chondral defect + meniscal tear	1.48 (0.57 to 3.83)	0.418	1.48 (0.57 to 3.83)	0.418
Meniscal repair	28 (19%)	11 (37.9%)	2.6 (1.1 to 6.11)	**0.029**
Partial meniscectomy	68 (46.3%)	14 (48.3%)	5.37 (0.72 to 39.78)	0.1
Osteochondral allograft	2 (1.4%)	2 (6.9%)	5.37 (0.72 to 39.78)	0.1
Other ligament reconstruction	11 (7.5%)	11 (37.9%)	7.56 (2.87 to 19.92)	** < 0.001**
HTO	1 (0.7%)	4 (13.8%)	23.36 (2.51 to 217.68)	**0.006**
DFO	1 (0.7%)	1 (3.4%)	5.21 (0.32 to 85.85)	0.248
Drilling/microfracture	2 (1.4%)	1 (3.4%)	2.59 (0.23 to 29.54)	0.444
Chondroplasty	15 (10.2%)	4 (13.8%)	1.32 (0.57 to 3.06)	0.525
ALL reconstruction	5 (3.4%)	1 (3.4%)	1.01 (0.11 to 9.02)	0.99

^a^Data are presented as mean (standard deviation) or *n* (%). Boldface *P* values indicate statistically significant difference between groups (*P* ≤ 0.05)

*ALL* anterolateral ligament, *CI* confidence interval, *DFO* distal femoral osteotomy, *HTO* high tibial osteotomy, *OR* odds ratio

### Complications

The time to complications after revision ACLR was not different for patients with a single-stage or a two-stage revision ACLR (Table 4). There is a significant association between patients who had a two-stage revision ACLR and anterior knee pain after surgery compared with patients who had single-stage revision ACLR (24.1% and 6.8% respectively, OR 4.36; 95% CI 1.5 to 12.65; *P* = 0.007). The SSI, stiffness, or symptomatic hardware rates did not differ between the revision cohorts. However, there was a significantly lower association of revision graft failure with patients who had a two-stage revision ACLR (3.4%) than patients who had a single-stage revision ACLR (23.1%) (OR 0.12, 95% CI 0.02 to 0.9; *P* = 0.04) (Table 4).

**Table 4:** Revision postoperative complications^a^

	One-stage (*n* = 147)	Two-stage (*n* = 29)	OR (95% CI)	*P* value
Time from revision to complication, days	750.3 (1026)	669.9 (514.2)	−80.4 (−553.19 to 392.39)	0.739
Anterior knee pain	10 (6.8%)	7 (24.1%)	4.36 (1.5 to 12.65)	**0.007**
SSI/wound dehiscence	1 (0.7%)	2 (6.9%)	10.81 (0.95 to 123.5)	0.055
Stiffness/arthrofibrosis	18 (12.2%)	6 (20.7%)	10.81 (0.95 to 123.5)	0.055
Symptomatic hardware	6 (4.1%)	1 (3.4%)	0.84 (0.1 to 7.24)	0.873
Graft failure	34 (23.1%)	1 (3.4%)	0.12 (0.02 to 0.9)	**0.04**

^a^Data are presented as mean (standard deviation) or *n* (%). Boldface *P* values indicate statistically significant difference between groups (*P* ≤ 0.05)

*CI* confidence interval, *OR* odds ratio, *SSI* superficial skin infection

### Matched analysis

In the matched analysis, 27 patients from the single-stage cohort were matched with 27 patients from the two-stage cohort. Many of the variables remained significant. However, an association between a higher BMI and two-stage revision ACLR (OR 3.47; 95% CI 0.59 to 6.35; *P* = 0.018) (Appendix) and use of autograft for two-stage revision ACLR (OR 8.62; 95% CI 1.6 to 46.45; *P* = 0.012) emerged.

### Patient reported outcomes

A total of 57 patients from the single-stage cohort completed the PROs, and 9 patients from the two-stage cohort completed the PROs (Table 5). The NPRS, KOOS Sport/Rec Score, KOOS quality of life (QOL) score, and VR-12 mental component score did not differ significantly between patients who had a two-stage revision ACLR or a single-stage revision ACLR. The revision cohorts did not vary significantly in return to sport rates (21.1% single-stage, 10.3% two-stage, OR 1.55; 95% CI 0.15 to 16.11; *P* = 0.714) or level of return to play (Table 5). Two-stage revision ACLR was significantly associated with a worse KOOS pain score (OR −11.7; 95% CI −22.35 to −1.04; *P* = 0.031), worse KOOS score (OR −17.11; 95% CI −28.85 to −5.36; *P* = 0.004) and worse KOOS Activities of Daily Living (ADL) score (OR −11.15; 95% CI −21.71 to −0.59; *P* = 0.039). Additionally, a lower VR-12 physical component score is significantly associated with a two-stage revision ACLR (OR −9.99; 95% CI −15.77 to −4.22; *P* = 0.001) (Table 5).

**Table 5:** Revision postoperative patient reported outcomes^a^

	One-stage (*n* = 57)	Two-stage (*n* = 9)	OR (95% CI)	*P* value
NPRS	2.4 [2]	3.8 (2.8)	1.46 (−0.29 to 3.2)	0.101
KOOS Sport/Rec Score	66.2 (4.4)	55 (24.9)	−11.23 (−28.19 to 5.73)	0.194
KOOS pain	83 [14]	71.3 (2.7)	−11.7 (−22.35 to −1.04)	**0.031**
KOOS score	74.2 (6.7)	57.1 (18.5)	−17.11 (−28.85 to −5.36)	**0.004**
KOOS ADL	89.1 (13.6)	77.9 (23.9)	−11.15 (−21.71 to −0.59)	**0.039**
KOOS QOL	58 (24.7)	42.4 (30.6)	−15.64 (−33.29 to 2.01)	0.082
VR-12 Mental Component score	50.8 (6.6)	40.8 (5.7)	−4.72 (−10.17 to 0.73)	0.089
VR-12 Physical Component Score	52.3 [8]	47.6 (6.8)	−9.99 (−15.77 to −4.22)	**0.001**
Return to sport	31 (21.1%)	3 (10.3%)	1.55 (0.15 to 16.11)	0.714
Same level	15 (10.2%)	2 (6.9%)	Referent	Referent
Better level	7 (4.8%)	1 (3.4%)	1.07 (0.08,13.9)	0.958
Worse level	9 (6.1%)	0	n/a	n/a

^a^Data are presented as mean (standard deviation) or *n* (%). Boldface *P* values indicate statistically significant difference between groups (*P* ≤ 0.05)

*ADL* activities of daily living, *CI* confidence interval, *KOOS* knee injury and osteoarthritis outcome score, *NPRS* numerical pain rating scale, *OR* odds ratio, *QOL* quality of life, *VR-12* Veteran RAND 12-Item Health Survey, *n/a* not applicable

### Multivariate analysis

The initial multivariate model contained the variables that were *P* ≤ 0.1 on bivariate, including two-stage revision, revision graft type, femoral and tibial tunnel position, ligamentous injury, HTO, revision femoral tunnel drilling technique, revision meniscal repair, and revision tibial and femoral metal screw fixation in predicting revision graft failure. After backward elimination variable reduction, the final reduced dependent model had two-stage revision ACLR and revision graft type (autograft versus allograft versus augmented allograft) as the significant factors relating to revision graft failure, as indicated by an LR with *P* ≤ 0.05. On multivariate analysis, the use of autograft in a revision procedure significantly reduced the risk of graft failure (OR 0.18; 95% CI 0.08 to 0.42; *P* < 0.001) as did the use of augmented allograft (OR 0.23; 95% CI 0.06 to 0.9; *P* = 0.035) (Table 6). Undergoing a two-stage revision, ACLR reduced the risk of graft failure by 85% and nearly reached significance (OR 0.15; 95% CI 0.02 to 1.17; *P* = 0.07).

**Table 6:** Multivariate analysis of revision graft failure

	OR (95% CI)	*P* value
Two-stage ACLR revision	0.15 (0.02 to 1.17)	0.07
Augmented allograft	0.23 (0.06 to 0.9)	**0.035**
Autograft	0.18 (0.08 to 0.42)	** < 0.001**

Boldface *P* values indicate statistically significant difference between groups (*P* ≤ 0.05)

*ACLR* anterior cruciate ligament reconstruction, *CI* confidence interval, *OR* odds ratio

## Discussion

The most important finding of this study was that two-stage revision ACLR is associated with a lower risk of graft failure but a higher risk of anterior knee pain and lower KOOS pain, KOOS Symptoms, KOOS ADL, and VR-12 Physical Component scores after primary ACLR failure. When predicting graft failure on multivariate regression, two-stage revision ACLR demonstrated an 85% lower risk of graft failure, which was insignificant.

This exhibits a significant departure from previous studies that found no significant differences in graft failure rate between single- and two-stage revision ACLR [20, 21, 23]. The study performed by Nielsen et al. had a larger number of patients undergoing a single- or two-stage revision ACLR after primary ACLR failure (sample size of 1331 and sample size of 243, respectively) [21]. It could be the larger sample size in that study is more reflective of the population. The present study also had a significantly higher use of autografts in the two-stage cohort compared with the single-stage cohort in the matched analysis. This likely reduced the risk of graft failure in the two-stage revision patients as it is well established in the literature that autograft use significantly lowers the risk of graft failure in revision ACLR [16, 24, 25]. This study also demonstrates a preference among included surgeons for the BPTB graft choice, particularly in the two-stage revision cohort. Within revision ACLR, the BPTB graft has demonstrated superior outcomes and been popularized as a suitable choice for athletes, consistent with this patient population. In a recent systematic review and meta-analysis, Chhabara et al. determined a lower failure rate among patients who received BPTB allografts relative to those who received tibialis anterior or Achilles allografts [26]. With respect to autografts, BPTB grafts have demonstrate superior failure rates to hamstring tendon and comparable results with the use of the quadriceps tendon in the setting of revision ACLR [27]. Gopinatth et al. determined that BPTB and hamstring autografts were most commonly used during the second-stage definitive reconstruction, lending credibility to the greater number of BPTB grafts used in the current study [28]. While the optimal graft choice within the realm of two-stage revision ACLR has not yet been fully investigated and is generally selected on the basis of surgeon preference, the greater preponderance of BPTB use in the two-stage revision population may also explain the observed difference in graft survival.

However, even after controlling for concomitant procedures and graft type in the single- and two-stage cohorts, two-stage revision ACLR was still a predictor in reducing graft failure rate in the multivariate model. Though this was not significant, it is possible that factors associated with a two-stage revision ACLR can result in less revision graft failure. The extended rehabilitation period and delayed return to the sport for two-stage revision patients could be associated with this reduced risk [11, 12, 16, 29]. Expanding the indications for two-stage revision ACLR to include young, competitive contact athletes could reduce their risk of revision graft failure. Further studies that compare revision graft failure rates between single-stage and two-stage cohorts are needed to better understand this association.

The significantly worse PROs for patients who underwent a two-stage revision ACLR after primary ACLR failure differ from previous studies that demonstrated no difference in PROs between single- and two-stage revision ACLR [20, 23]. However, due to the study’s retrospective nature, less than 40% of the patients reported the PROs. This makes it difficult to draw conclusions on them. The worse KOOS pain scores in the two-stage cohort could be related to this group’s increased risk of anterior knee pain. Anterior knee pain may be due to graft donor site pain from harvesting the patellar tendon autograft in the two-stage cohort [30–32], which was the majority graft type used in the two-stage procedure in this study. The additional surgeries for the two-stage cohort may also relate to increased knee pain due to repeated surgical trauma [11]. Importantly, anterior knee pain has been associated with a number of causal etiologies following ACLR and, therefore, we cannot conclusively comment on whether this relationship is directly due to the two-stage procedure, greater use of BPTB, or another etiology such as chondromalacia of the patella or a patellar chondral lesion, among others. Additionally, the significantly increased incidence of concomitant injuries and procedures reported in the two-stage cohort can cause decreased activity and sports performance in the two-stage cohort [11, 33, 34].

Surprisingly, even with controlling for BMI in the matched analysis between patients who had single- or two-stage revision ACLR after primary ACLR failure, there was a significant association of a higher BMI in patients undergoing a two-stage revision. This might be due to tunnel widening during graft failure, as patients with elevated BMI have a higher incidence of low-velocity graft failure [35–37], leading to a staged procedure to bone graft the widened tunnels. This study was unable to quantify tunnel diameter due to the retrospective nature of data collection that inconsistently reported tunnel diameter and the lack of standardized CT scan evaluation for tunnel expansion. The influence of elevated BMI and tunnel diameters after graft failure should be investigated further.

There was a higher association between the utilization of the hybrid transtibial (HTT) femoral tunnel drilling approach and the use of metal interference screws for graft fixation in the two-stage cohort. Surgeon preference is likely responsible for this difference, which did not significantly affect graft failure rates. The preference for patellar tendon graft in the two-stage cohort also reflects the increased utilization of the metal interference screw, as this was the fixation of choice for this graft. Similarly, this did not affect the graft failure rate.

There are inherent differences in the indications for a single-stage versus two-stage revision ACLR procedure. The present study found a significant association in tunnel malposition for patients with a two-stage revision compared with a single-stage, as well as a significant increase in osteotomy, multiligamentous procedures, and meniscal procedures. This is expected, as a two-stage revision ACLR is needed to correct mispositioned tunnels [38]. Our study also utilized two-stage revision ACLR when additional alignment or multiligamentous were needed, further broadening the indication for two-stage procedures from its previous tunnel-based restrictions [29]. Still, the fundamental distinction in who should undergo a two-stage revision rather than a single-stage revision remains, creating unequal cohorts for comparison.

This study has limitations, particularly with regard to its retrospective design. Relying on chart review for data collection creates a less reliable source than a prospective collection due to incomplete data and, therefore, introduces possible selection bias. In addition, there is potential for confounders; these results do not signify causation, only association. The large time span of eligible patients limited the collection of patient-reported outcomes, as many patients who had surgery over 10 years ago, despite having the required follow-up, were not responsive when contacted to complete the PRO questionnaires limiting the generalizability of the study findings. Consequently, additional surgeries or complications could not be reported in the study because of the inability to contact all eligible patients.

## CONCLUSION

In this study, objective outcomes and subjective patient scores significantly differed between single-stage and two-stage revision ACLR after primary ACLR failure. Patients with a two-stage revision ACLR had significantly reduced risk of revision graft failure. Still, two-stage revision ACLR patients had higher rates of postoperative anterior knee pain, worse reported pain scores, and lower knee function than single-stage patients. Undergoing a two-stage revision causes a significant surgical burden on these patients, so shared decision-making of expected outcomes should occur with appropriate patient selection. Surgeons should further consider balancing the potential risk of graft failure in single-stage revision with the risk of lower knee function over time with the two-stage procedure. Further longitudinal studies are recommended to compare single-stage and two-stage revision ACLR outcomes after primary ACLR failure.

## Data Availability

The datasets generated and/or analyzed during the current study are not publicly available as they are part of a proprietary database but are available from the corresponding author on reasonable request.

## Abbreviations

ACL: Anterior cruciate ligament
ACLR: ACL reconstruction
SSI: Superficial skin infection
VTE: Venous thromboembolism
PRO: Patient-reported outcome
NPRS: Numerical pain rating scale
KOOS: Knee Injury and Osteoarthritis Outcome Score
VR-12: Veterans RAND 12-Item Health Survey
BMI: Body mass index
HTT: Hybrid transtibial
CI: Confidence interval
OR: Odds ratio
HTO: High tibial osteotomy
DFO: Distal femoral osteotomy
ALL: Anterolateral ligament
QOL: Quality of life
